# A Treatise on the Diseases and Physical Education of Children

**Published:** 1850-11

**Authors:** 


*A Treatise on the Diseases and Physical Education of Children.* By JOHN EBERLE, M. D., late Prof. of the Theory and Practice of Medicine in Transylvania University, etc., etc., etc. Fourth edition, with notes and large additions by THOMAS D. MITCHELL, A. M., M. D., Prof. of the Theory and Practice of Medicine in the Philadelphia College of Medicine, etc., etc., etc. Philadelphia: Lippincott, Grambo & Co., successors to Grigg, Elliot & Co., No. 14 North Fourth-street. 1850. 8 vo., pp. 768.

*Eberle on Children* not many years since was a standard work on infantile diseases. It is now, for the most part, supplanted by treatises which are more in conformity with the present stage of the progress of practical medicine. There is, however, much contained in that work which is valuable, and while it cannot be recommended as a text book, it may be read with advantage by the practitioner who is familiar with the modern literature of the diseases of children. To adapt it to the existing condition of medical science Prof. Mitchell has appended a Sequel of over three hundred pages. The latter has also been published separately for the convenience of those who already possess the former editions of Eberle's work. For sale by Phinney & Co.

